# Placental epigenetic age and adolescent blood pressure: the Extremely Low Gestational Age Newborn cohort

**DOI:** 10.1038/s41390-025-04110-0

**Published:** 2025-05-07

**Authors:** Anisha Gerber, Kyle R. Roell, Katelyn K. Huff, Thomas Michael O’Shea, Rebecca C. Fry, Keia Sanderson

**Affiliations:** 1 Division of Pediatric Nephrology, Department of Pediatrics, University of Virginia, Charlottesville, VA USA; 2 Institute for Environmental Health Solutions, Gillings School of Global Public Health, The University of North Carolina at Chapel Hill, Chapel Hill, NC USA; 3 The University of North Carolina at Chapel Hill Gillings School of Global Public Health, Department of Environmental Sciences and Engineering, Chapel Hill, NC USA; 4 Division of Neonatology, Department of Pediatrics, The University of North Carolina at Chapel Hill, Chapel Hill, NC USA; 5 Department of Medicine-Nephrology, The University of North Carolina at Chapel Hill, Chapel Hill, NC USA

## Abstract

**Background:**

We examined the association between placental epigenetic gestational age (eGA) acceleration and adolescent systolic blood pressure (SBP) in a cohort born extremely preterm.

**Methods:**

Study participants were a subset of the Extremely Low Gestational Age Newborn cohort (born <28 weeks’ gestation) who had placental DNA methylation quantified and had SBP measured during adolescent follow-up. eGA acceleration was calculated as the residual from the regression of predicted placental eGA (using the Robust Placental Clock) onto chronological gestational age. Unadjusted and adjusted mixed effects models were used to test the association between eGA acceleration and adolescent SBP. We also tested the interaction of eGA acceleration and sex on SBP.

**Results:**

In the overall sample (*N* = 193), we found no association between placental eGA acceleration and adolescent SBP. When interaction between eGA acceleration and sex was tested, males had a 3.6 mmHg increase in SBP (95% CI 0.9, 6.4; *p* = 0.01) for every 1-week acceleration in eGA after adjusting for confounders.

**Conclusion:**

Placental eGA acceleration is associated with SBP increase in adolescent males but not females born extremely preterm, supporting the hypothesis that placental eGA could be evaluated as a risk biomarker for childhood cardiovascular outcomes.

**Impact:**

- This study examines the association between placental epigenetic gestational age (eGA) and adolescent blood pressure. For every 1-week acceleration in placental eGA, adolescent males born extremely preterm had a 3.6 mmHg increase in systolic blood pressure (95% CI 0.9, 6.4; *p* = 0.01) after adjusting for confounders. The same association was not seen in females or the overall cohort.
- Our sex-specific finding supports the hypothesis that differences in placental eGA are associated with childhood health.
- Placental eGA estimation as a tool for identifying children who are at risk for developing elevated blood pressure should be further evaluated in other cohorts.

## Introduction

Epigenetic age is a biological age estimator that reflects a wide variety of physiologic processes from cell differentiation to tissue homeostasis.^1,2^ Based on DNA methylation across the genome, epigenetic age is especially salient for multi-organ disease processes such as hypertension (HTN).^3^ In adults, epigenetic age in excess of chronological age is associated with HTN and holds potential as a biomarker to identify people at higher risk for cardiovascular (CV) and kidney disease.^4–6^ In children, there is growing evidence of association between gene-specific DNA methylation differences at birth and systolic blood pressure (SBP) later in childhood.^7,8^ However, there are few studies to date that evaluate the association between epigenetic age at birth and longer-term blood pressure (BP), kidney, or CV outcomes.^9,10^

Adults born extremely preterm are at increased risk for CV and kidney diseases as well as all-cause mortality.^11–13^ Male adults born extremely low birth weight exhibit accelerated epigenetic aging, but it is not known whether accelerated epigenetic aging contributes to the higher risk of chronic illness among individuals born extremely preterm.^14^ The developmental origins of health and disease (DOHaD) conceptual model posits that early life exposures, including fetal exposures, can contribute to the risk of chronic illnesses manifesting decades after birth.^15–17^ In support of this model, characteristics of the placenta, such as histological features, DNA methylation of specific loci, and microbiological characteristics, have been associated with health outcomes in the offspring.^18–24^

However, we are aware of no studies on whether long-term health outcomes are related to epigenetic age acceleration in the placenta. Epigenetic age acceleration in the placenta is of particular interest because it has been associated with maternal health conditions, such as preeclampsia, and environmental exposures, such as maternal smoking.^25–28^ Metabolic constraints passed to the fetus via epigenetic signaling in the placenta can affect organogenesis, including nephron development, and dysregulated organogenesis could have lifelong impacts on BP.^15,17^

Our aim was to study the association between placental epigenetic gestational age (eGA) acceleration and adolescent BP. We used data from the Extremely Low Gestational Age Newborn (ELGAN) cohort, which was enrolled at birth and followed through adolescence. All members of the cohort were born before 28 weeks of gestation and thus constitute a group with increased risk of CV and kidney disease.^11,12^ Our primary health outcome was SBP because it is a risk factor for progressive CV and kidney disease. Because sex differences in placental epigenetics and epigenetic aging over the lifespan have been observed, we analyzed the association between placental eGA at birth and adolescent SBP in both the overall sample as well as by sex.^14,29^

## Methods

### Study population

This is a secondary analysis of the ELGAN study, the details of which have been previously described.^30^ Briefly, the ELGAN study is a multicenter cohort of 1506 newborns born prior to 28 weeks between 2002 and 2004 across 14 hospitals in the United States. Written parental consent for mother and newborn enrollment was obtained shortly before birth or soon after delivery. Of the enrolled newborns, placental DNA methylation was quantified on a subset who had sufficient placental tissue available and returned for follow-up at 10 years old. Our sampling frame consisted of participants who had placental DNA methylation quantified and had BP prospectively measured during a follow-up study visit between 15 and 18 years old (2017–2020). All study procedures were approved by an institutional review board at each site participating in this multicenter study.^30,31^

### Measurement of placental eGA acceleration

The measured exposure was placental eGA acceleration at birth. Chronological gestational age in the ELGAN cohort was based on date of embryo retrieval, intrauterine insemination, or ultrasound before the 14th week of gestation when possible (62%). When this information was unavailable, estimations were based on later ultrasound (29%), menstrual dating (7%), or gestational age recorded in the medical chart (1%).^26^

Each infant had a placental sample collected from chorionic tissue shortly after birth using a standardized procedure that has previously been described.^26,29^ The samples were immediately frozen and stored at −80 °C until shipment to the University of North Carolina at Chapel Hill for processing, methylation profiling, and computation of the ratio of methylated to unmethylated signal intensities (β-values).^26,29^

Placental eGA was calculated using the Robust Placental Clock (RPC), which was trained on placental data from pregnancies ranging from 5 to 42 weeks’ gestation and consists of placental DNA methylation β-values from 558 CpG sites. The RPC is considered unaffected by pregnancy complications (e.g., preeclampsia) because placentas from such pregnancies were included in its construction. The RPC training and validation sets are representative of preterm births that are commonly precipitated by pregnancy complications.^32^

To derive eGA acceleration, either age acceleration difference or age acceleration residual can be used. The mean observed epigenetic age, and therefore the age acceleration difference, shifts based on preprocessing methods. We used residuals because they are derived relative to the measured sample and are more comparable across sample sets.^33,34^ Age acceleration residuals were extracted from a linear regression model of RPC-predicted placental eGA on chronological gestational age. Positive values represent acceleration, and negative values represent deceleration. These techniques have also been described in detail elsewhere.^26,29^

### SBP measurement

The primary outcome was average SBP measurement, prospectively collected either at the 15-year study visit or at the study visit between 17 and 18 years old. For the 15-year visit, parents provided consent and adolescents provided assent specifically for BP measurement, which was an ancillary component of the ELGAN study. The visit between 17 and 18 years old was a part of the larger National Institutes of Health Environmental Influences on Childhood Health Outcomes (ECHO) study, for which the initial ELGAN ECHO consent and assent encompassed BP measurement.

No participants were taking antihypertensive medications at the time of their adolescent study visits, based on information provided by a parent or guardian. Each participant had one to three oscillometric BPs prospectively measured on the right upper extremity with an appropriately sized cuff. Participants were seated with their feet flat on the floor. The first BP was measured after five minutes of rest, and each subsequent BP was measured five minutes apart. The SBP values were averaged to obtain a final measurement.^31,35^

Average SBP measurement for each participant was recorded as a continuous variable and analyzed as both a continuous and a binary variable, defined as elevated SBP ≥ 120 mmHg or normal SBP < 120 mmHg per the 2017 American Academy of Pediatrics (AAP) guidelines.^36^ For preadolescents, diagnosis of elevated BP or HTN is based on percentiles determined by age, sex, and height, but for adolescents, the AAP guidelines recommend a standard cutoff of SBP ≥ 120 mmHg in accordance with American Heart Association guidelines.^36,37^ We focused on SBP because it is a strong predictor of adult HTN and CV disease.^38–40^

### Identification of covariates

Maternal, neonatal, and childhood covariates were selected based on prior studies indicating a relationship between certain characteristics and neonatal and childhood CV and kidney outcomes.^26,31^ Maternal covariates of health insurance, years of education, marital status, age, height and weight (from which body mass index was calculated), and prenatal smoking, as well as infant race, were self-reported at the time of ELGAN enrollment. Covariates recorded from the medical chart at time of enrollment included maternal diabetes and HTN, as well as neonatal sex, weight, and chronological gestational age at birth.^26^ Adolescent body mass index (BMI) was calculated from measurements of height and weight at the adolescent study visit between 15 and 18 years old.^41^ Diastolic BP (DBP) was also measured at the adolescent study visit. Asthma diagnosis during adolescence was based on a parent or guardian's verbal report.

Covariates were analyzed using a directed acyclic graph (DAG) to identify confounding variables (Supplementary Fig. S1).^42^ The minimal sufficient adjustment set, as identified by the DAG, includes maternal factors (health insurance, years of education, relationship status, prenatal smoking, pre-pregnancy BMI > 30 kg/m^2^, diabetes, and hypertension), participant sex, and neonatal birth weight for gestational age, which we regard as an indirect measure of fetal growth.^43,44^ Adolescent BMI and asthma diagnosis were reported as covariates but not identified as confounders.

For a parsimonious model, we combined the maternal factors of private or other health insurance (yes = 1, no = 0), years of education (more than high school = 1, high school graduate or less = 0), and marital status (married or living with partner = 1, not married or living with partner = 0) into a previously described composite indicator of socioeconomic position (SEP).^26,45^ The summative score was assessed as a categorical variable ranging from 0 to 3, with higher scores indicating higher SEP. Previous studies in the ELGAN population found SEP to be predictive of altered placental DNA methylation.^26,45^

### Statistical analysis

Descriptive statistics for adolescents in our sample were stratified by elevated SBP measurement (≥120 mmHg) versus normal SBP measurement (<120 mmHg) and included frequencies with percent for categorical variables and mean with standard deviation (SD) for normally distributed continuous variables. For BP, we reported both mean (SD) because it was normally distributed and median (IQR) to provide more detail regarding our outcome variable. We reported the range for epigenetic age acceleration. We also reported descriptive statistics for surviving adolescents who completed ELGAN follow-up between 15 and 18 years old, but are not included in our sample.

Mixed effects linear regression, using random effects to account for relatedness of study participants (e.g., twins), was used to evaluate the association between placental eGA acceleration and adolescent SBP as a continuous variable. We then adjusted the mixed linear model for DAG-identified confounders: maternal factors (SEP, prenatal smoking, pre-pregnancy BMI > 30 kg/m^2^, diabetes, and hypertension), participant sex, and neonatal birth weight for gestational age. Estimates from the models are reported as SBP differences in mmHg with 95% confidence intervals (CIs) and *p*-values.

The placental chorion is comprised of multiple cell types. Thus, for sensitivity analysis, the unadjusted and adjusted mixed linear models were fit with placental cell-type proportions, estimated by the R package *planet*, as covariates.^46^

Mixed effects logistic regression was used to measure the association between placental eGA acceleration and the dichotomized outcome of average SBP measurement ≥120 mmHg or <120 mmHg. The model was then adjusted for the previously mentioned DAG-identified confounders. We reported odds ratios with 95% CIs and *p*-values for associations between placental eGA acceleration and elevated adolescent SBP.

Finally, given known sex differences in placental epigenetics and epigenetic aging, we performed interaction analyses to evaluate sex as an effect measure modifier.^14,29,47^ We tested the interaction between sex and placental eGA acceleration on adolescent SBP in unadjusted and adjusted mixed linear and logistic regression models. To further test the interaction, we ran an adjusted mixed linear regression controlling for adolescent BMI > 30 kg/m^2^ and another controlling for asthma in adolescence, which are both covariates associated with adverse CV outcomes.^48,49^

Statistical significance was defined as a two-sided *p*-value < 0.05. All analyses were conducted with R Core Team (2023).

## Results

### Study population

**Fig. 1:**
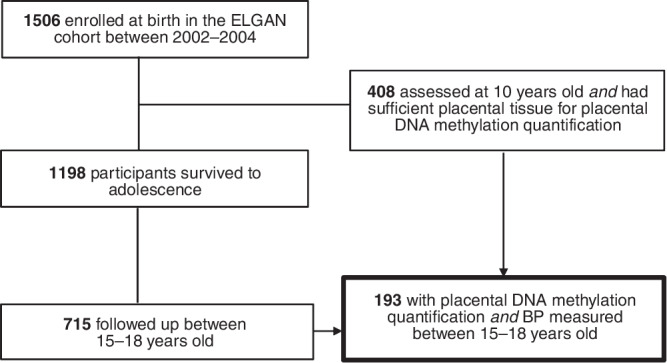
Flow diagram for this secondary analysis of the Extremely Low Gestational Age Newborn (ELGAN) study, illustrating our sampling frame (*N* = 193) in the context of all participants enrolled in the ELGAN study (*N* = 1506).

Of 715 ELGAN participants who completed adolescent follow-up, 193 (27.0%) had placental DNA methylation quantified and had SBP measured at their adolescent study visits (Fig. 1). Of these 193 participants, 72 (37.3%) had elevated SBP at their adolescent follow-up visit (Table 1). Ninety-three (48.2%) were female: 33.3% of the elevated SBP group versus 57.0% of the normal SBP group. One hundred (51.8%) were male: 66.7% of the elevated SBP group versus 43.0% of the normal SBP group. Adolescents with elevated SBP had a higher proportion of mothers with pre-pregnancy BMI > 30 kg/m^2^ (27.8%) compared to adolescents with normal SBP (19.0%). Adolescents with elevated SBP also had a higher proportion of mothers with hypertension (25.0%) compared to adolescents with normal SBP (14.9%).

**Table 1:** Neonatal, childhood, and maternal characteristics of participants stratified by elevated versus normal SBP measurement at 15–18 years old.

	Elevated SBP ($\ge$120 mmHg) *N* = 72	Normal SBP ( < 120 mmHg) *N* = 121	Total *N* = 193
Maternal characteristics; *n* (%) or mean (SD)			
Age at delivery (years), mean (SD)	29.5 (6.7)	29.6 (7.1)	29.5 (7.0)
BMI (kg/m^2^)			
Underweight (<18.5)	2 (2.8%)	11 (9.1%)	13 (6.7%)
Normal (18.5 to <25)	30 (41.7%)	65 (53.7%)	95 (49.2%)
Overweight (25 to <30)	17 (23.6%)	19 (15.7%)	36 (18.7%)
Obese (≥30)	20 (27.8%)	23 (19.0%)	43 (22.3%)
Missing	3 (4.1%)	3 (2.5%)	6 (3.1%)
Education Status			
Less than a high school diploma	7 (9.7%)	16 (13.2%)	23 (11.9%)
High school diploma	22 (30.6%)	27 (22.3%)	49 (25.4%)
Some college	17 (23.6%)	27 (22.3%)	44 (22.8%)
Bachelor’s degree with or without postgraduate education	25 (34.7%)	49 (40.5%)	74 (38.3%)
Missing	1 (1.4%)	2 (1.7%)	3 (1.6%)
Public insurance			
Yes	25 (34.7%)	38 (31.4%)	63 (32.6%)
No	47 (65.3%)	83 (68.6%)	130 (67.4%)
Relationship status			
Married or living with a partner	56 (77.8%)	101 (83.5%)	157 (81.3%)
Not married or living with a partner	16 (22.2%)	20 (16.5%)	36 (18.7%)
Prenatal smoking			
Yes	6 (8.3%)	18 (14.9%)	24 (12.5%)
No	65 (90.3%)	103 (85.1%)	168 (87.0%)
Missing	1 (1.4%)	0 (0%)	1 (0.5%)
Diabetes, including gestational, type 1, and type 2			
Yes	6 (8.3%)	10 (8.3%)	16 (8.3%)
No	65 (90.3%)	111 (91.7%)	176 (91.2%)
Missing	1 (1.4%)	0 (0%)	1 (0.5%)
Hypertension, including gestational and chronic			
Yes	18 (25.0%)	18 (14.9%)	36 (18.7%)
No	53 (73.6%)	103 (85.1%)	156 (80.8%)
Missing	1 (1.4%)	0 (0%)	1 (0.5%)
Participant (adolescent) characteristics; *n* (%), mean (SD), median (IQR), or range			
Sex			
Male	48 (66.7%)	52 (43.0%)	100 (51.8%)
Female	24 (33.3%)	69 (57.0%)	93 (48.2%)
Race			
White	44 (61.1%)	81 (66.9%)	125 (64.8%)
Black	24 (33.3%)	28 (23.2%)	52 (26.9%)
Other	4 (5.6%)	12 (9.9%)	16 (8.3%)
Adolescent BMI (kg/m^2^)			
≤30	54 (75.0%)	100 (82.6%)	154 (79.8%)
>30	17 (23.6%)	8 (6.6%)	25 (13.0%)
Missing	1 (1.4%)	13 (10.8%)	14 (7.2%)
Adolescent asthma diagnosis			
Yes	18 (25.0%)	27 (22.3%)	45 (23.3%)
No	53 (73.6%)	82 (67.8%)	135 (70.0%)
Missing	1 (1.4%)	12 (9.9%)	13 (6.7%)
Gestational age (weeks), mean (SD)	26.1 (1.3)	26.1 (1.3)	26.1 (1.3)
Birth weight-for-gestational-age *z* score			
<−2	1 (1.4%)	7 (5.8%)	8 (4.1%)
−2 to <−1	8 (11.1%)	11 (9.1%)	19 (9.9%)
−1 to 1	54 (75.0%)	90 (74.4%)	144 (74.6%)
>1	9 (12.5%)	13 (10.7%)	22 (11.4%)
Epigenetic age acceleration (weeks), mean (SD)	−0.03 (0.80)	0.06 (1.1)	0.03 (1.0)
Epigenetic age acceleration range (weeks)	−1.7, 1.9	−2.4, 6.8	−2.4, 6.8
Adolescent BP (mmHg)			
SBP, mean (SD)	131 (10.4)	110 (6.7)	118 (13.0)
SBP, median (IQR)	127.5 (13.2)	111 (9.7)	116 (16)
DBP, mean (SD)	78.1 (8.5)	69.4 (8.30)	72.6 (9.4)
DBP, median (IQR)	79.2 (11.3)	69.3 (10)	72.3 (12.3)

Participants had a mean chronological gestational age at birth of 26.1 weeks (SD 1.3 weeks), and the majority (74.6%) had birth weight within 1 SD of the mean for their gestational age. Mean placental eGA deviation was less than one day from chronological gestational age (SD 7 days). Placental eGA acceleration ranged widely from −2.4 weeks to 6.8 weeks. Characteristics between adolescents in our sampling frame (*N* = 193) and those not in our sampling frame (*N* = 522) were similar, including sociodemographic characteristics (Supplementary Table S1).

### Epigenetic age acceleration at birth and SBP in adolescence

In the overall sample, placental eGA acceleration at birth was not associated with adolescent SBP in the unadjusted mixed linear model (95% CI −1.4, 2.1, *p* = 0.65; Table 2). Additionally, there was no association between placental eGA acceleration at birth and adolescent SBP in the mixed linear model adjusted for covariates identified as confounders by the DAG (95% CI −1.0, 2.4, *p* = 0.45). Similar results were obtained in the sensitivity analysis that controlled for cell-type proportions (Supplementary Table S2).

**Table 2:** Mixed linear regression of placental eGA acceleration on adolescent SBP.

	Β Coefficient	95% CI	*P*-value
Unadjusted model	0.4	−1.4, 2.1	0.65
Adjusted model^a^	0.7	−1.0, 2.4	0.45
Interaction between eGA acceleration and sex			
Unadjusted model interaction	3.8	0.27, 7.3	0.04
Males	2.8	0.03, 5.5	0.05
Females	−1.0	−3.2, 1.2	0.37
Adjusted model interaction^b^	4.7	1.3, 8.1	0.01
Males	3.6	0.9, 6.4	0.01
Females	−1.1	−3.2, 1.0	0.34

^a^adjusted for SEP, prenatal smoking, maternal BMI > 30 kg/m^2^, maternal diabetes, maternal hypertension, birth weight for gestational age, and participant sex.

^b^adjusted for SEP, prenatal smoking, maternal BMI > 30 kg/m^2^, maternal diabetes, maternal hypertension, and birth weight for gestational age.

**Fig. 2:**
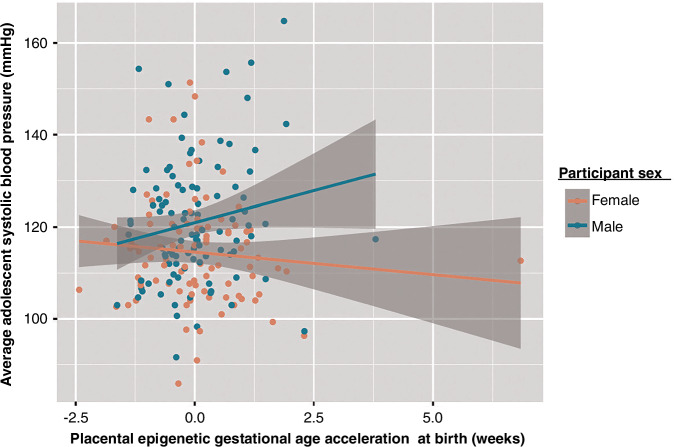
Scatterplot of placental eGA acceleration and adolescent SBP stratified by sex. Pearson correlation coefficient (*r*) = 0.157 (male) and −0.098 (female).

In the unadjusted interaction analysis of sex and placental eGA on adolescent SBP as a continuous variable, the interaction coefficient was 3.8 (95% CI 0.27, 7.3, *p* = 0.04; Table 2), with an SBP increase of 2.8 mmHg for every 1-week increase in placental eGA compared to chronological gestational age for males (95% CI 0.03, 5.5, *p* = 0.05). Adjusting for confounders, the interaction coefficient was 4.7 (95% 1.3, 8.1, *p* = 0.01). For every 1-week increase in placental eGA compared to chronological gestational age, SBP increased by 3.6 mmHg for males (95% CI 0.9, 6.4, *p* = 0.01) after adjusting for confounders (Fig. 2). This interaction remained even after adjustment for cell-type proportions (95% CI 1.4, 7.1, *p* = 0.01; Supplementary Table S2). As shown in Supplementary Table S3, the interaction also remained after controlling for adolescent BMI > 30 kg/m^2^ (95% CI 0.6, 5.9, *p* = 0.02) and asthma (95% CI 0.9, 6.4, *p* = 0.02). No association was seen for females.

In the overall sample, placental eGA acceleration was not associated with adolescent SBP as a dichotomous variable in the unadjusted (95% CI 0.7, 1.2, *p* = 0.53) or adjusted (95% CI 0.6, 1.3, *p* = 0.58) mixed models (Table 3). No interaction was seen between sex and placental eGA acceleration on SBP as a dichotomous outcome in the unadjusted (95% CI 0.8, 3.3, *p* = 0.23) or adjusted (95% CI 0.9, 4.5, *p* = 0.13) mixed models.

**Table 3:** Mixed logistic regression of placental eGA acceleration and elevated adolescent SBP.

	Odds ratio (95% CI)	*P*-value
Unadjusted model (ref = normal SBP)	0.9 (0.7, 1.2)	0.53
Adjusted model (ref = normal SBP)^a^	0.9 (0.6, 1.3)	0.58
Interaction between eGA acceleration and sex		
Unadjusted model interaction (ref = normal SBP)	1.6 (0.8, 3.3)	0.23
Males	1.1 (0.7, 1.8)	0.65
Females	0.7 (0.4, 1.2)	0.22
Adjusted model interaction (ref = normal SBP)^b^	1.9 (0.9, 4.5)	0.13
Males	1.3 (0.8, 2.3)	0.40
Females	0.7 (0.4, 1.2)	0.19

^a^adjusted for SEP, prenatal smoking, maternal BMI > 30 kg/m^2^, maternal diabetes, maternal hypertension, birth weight for gestational age, and participant sex.

^b^adjusted for SEP, prenatal smoking, maternal BMI > 30 kg/m^2^, maternal diabetes, maternal hypertension, and birth weight for gestational age.

## Discussion

To our knowledge, this study is the first to investigate the relationship between placental eGA acceleration and adolescent BP in those born extremely preterm. In our overall sample, we found no association between placental eGA and adolescent SBP. Interestingly, placental eGA acceleration at birth was associated with an SBP increase in adolescent males but not females born extremely preterm. Among males, after adjusting for confounders, the estimated increase in SBP was 3.6 mmHg for each week that placental eGA exceeded chronological gestational age. If externally validated, the sex-specific association between placental eGA acceleration and adolescent SBP in our sample of individuals born extremely preterm would support the possibility that placental epigenetics and resultant fetal programming influence cardiometabolic and kidney disease throughout the life course, consistent with the hypothesis of developmental origins of chronic disease.^50^

The early life exposome, including maternal diet, dyslipidemia, preeclampsia, and gestational diabetes, is associated with placental epigenetic aging, and there is mounting evidence that accelerated placental epigenetic age is associated with preterm birth.^51–56^ However, the association between placental aging and childhood health outcomes (including HTN, for which children born preterm are at higher risk) remains largely unexplored.^57–59^ Although sexual dimorphism in the placenta has been described in response to perinatal stress, sexual dimorphism in the association between placental eGA acceleration and SBP in adolescents born extremely preterm has not previously been reported.^29,47,60^

### No association between eGA acceleration and adolescent SBP in an at-risk sample

Placental epigenetic signaling has been associated with altered fetal development and organogenesis, which could influence later life health outcomes.^18^ Given the key role of DNA methylation in kidney development, perinatal epigenetic signaling may have a lasting influence on BP by altering nephrogenesis.^61,62^ However, in contrast to prior studies that focused on epigenetic age in adult blood samples and umbilical cord blood and on gene-specific DNA methylation changes in umbilical cord blood, we did not find evidence in the overall sample to support the association between placental eGA acceleration at birth and elevated SBP among extremely preterm born adolescents.^7,8,10,63^

Differences between our study and previous studies that may explain divergent findings include our use of the RPC instead of cord blood-based DNA methylation measures.^7,9,10^ The kidney plays an important role in the regulation of arterial BP, and differential epigenetic patterning in genes for transcriptional regulators, including Homeobox (*HOX)* and Paired box (*PAX)* genes, plays a major role in early kidney development.^64^ There may be a subset of gene-specific epigenetic modifications within the 558 DNA methylation sites of the RPC that have stronger associations with CV and kidney outcomes.^32,61^

In a study of 422 young adults born before 32 weeks’ gestation, 10% had elevated BP.^57^ In comparison, 37.3% of our participants born before 28 weeks’ gestation had elevated BP, which may also contribute to the divergent findings between our study and others that reported an association between epigenetic changes, mostly at term or late preterm birth, and childhood BP.^7,8,10^

The cohort that we studied is not enriched in risk factors for childhood elevated BP, such as mothers with pre-pregnancy obesity or adolescents with obesity, or a history of fetal growth restriction.^35,49,65–67^ However, the relatively high proportion of adolescents with elevated SBP measurements that we observed in the ELGAN cohort is consistent with the frequently observed increase in adverse health outcomes and mortality with increasing degree of prematurity.^11–13^ Nearly 60% of nephrons are formed during the second and third trimesters of gestation, and total nephron number correlates with gestational age at birth.^68–70^ Infants born extremely preterm are more likely to have dysregulated nephrogenesis and reduced nephron number, both of which increase their lifetime risk for HTN.^31,71,72^

None of the adolescents in our sample were previously prescribed antihypertensive medications, indicating that none had a prior diagnosis of HTN, but pediatric HTN is underdiagnosed.^36,73–75^ ELGAN adolescents are a high-risk group for HTN due to their extreme prematurity. Extreme prematurity is likely a prominent contributor to the higher proportion of elevated BP measurements in our study and highlights the potential relevance of focusing on epigenetic changes in hypertension-related genes at extremely preterm birth.^15^

### Sex as an effect measure modifier

Sex has been found to modify associations between placental epigenetic aging and fetal growth and between epigenetic age in umbilical cord blood and childhood blood pressure.^10,47^ Similarly, in our interaction analysis of sex and placental eGA acceleration on adolescent SBP, the beta coefficients for males and females differed from each other (3.6 and −1.1, respectively) and from the beta coefficient for the adjusted model in the overall sample (0.7), supporting sex as an effect measure modifier.^76^ Estimates of the association between placental eGA acceleration and adolescent SBP for males and females were sufficiently different from each other to suggest that extremely preterm-born males are particularly susceptible to higher SBP in the setting of placental eGA acceleration. However, our finding that sex modifies the association between placental eGA and adolescent SBP requires validation in other pediatric cohorts.

Adult men but not adult women born with extremely low birth weight have older epigenetic age compared to their peers born at normal birth weight, and there is growing evidence of sex-dependent epigenetic signaling even at birth in vulnerable groups of neonates.^14^ Sex-dependent epigenetic patterning in the placenta has been described in genes encoding immune function, transcription factor signaling, and cellular transmembrane transport.^29^ Sexual dimorphism is also seen in fetal growth, in which placental eGA acceleration is associated with reduced fetal growth in males but not in females.^47^ Fetal growth restriction, in turn, is associated with impaired nephrogenesis, HTN, and CV disease.^47,77,78^ Sex-dependent associations between placental eGA acceleration and neonatal health outcomes have been attributed to a combination of mechanisms, including higher placental global DNA methylation and tighter regulation of gene expression in females that may protect against negative effects of epigenetic age acceleration.^47,79^

Our sex-specific finding contributes to the growing body of literature supporting the theory of developmental origins of health and disease, which links adverse fetal development and long-term health.^50^ Our study also provides support for the possibility that biological age estimators might be useful as clinical predictors of chronic disease, such as pediatric chronic kidney disease, which is closely linked to CV morbidity and in which early intervention with lifestyle modifications and medication slows disease progression.^80–82^ Whether the physiologic stressors that cause epigenetic aging or epigenetic aging itself drives disease, epigenetic age may be useful as a biomarker for risk stratification in pediatric cohorts at high risk for CV and kidney disease.^3,83–85^ Epigenetic age may also be a useful biomarker to follow over time in clinical care and research because it can be slowed or hastened by variables such as diet, exercise, and psychological stress.^86,87^

### Strengths and limitations

A strength of this study is the use of placental tissue to estimate eGA via the Robust Placental Clock. The RPC has a high correlation (*r* = 0.99) between predicted and chronological age in its validation set, which minimizes measurement error seen with newer and less validated biological age estimators.^26,32^ Placental eGA provides distinct information from cord blood age, and this study is among the first to evaluate the association between placental eGA and adolescent health.^10,88^ Use of placental tissue, as opposed to cord blood, focuses on DNA methylation at the interface between maternal and fetal circulations. Compared to cord blood, placental epigenetic age might be more reflective of maternal exposures during pregnancy, providing more information about how maternal exposures and health impact the fetus during gestation.

Similarly, ELGAN longitudinal data is a strength because it provides insight into how perinatal epigenetics relate to health outcomes after more than a decade of growth and childhood development. The rate of change in epigenetic age is exponential in childhood and adolescence, after which it is linear.^89^ A limitation of this cohort is the lack of data on markers of neonatal kidney health, so the relationship between neonatal kidney health, epigenetic age, and adolescent BP cannot be evaluated. In future research of preterm cohorts, the collection of data on neonatal kidney markers could provide valuable information on the origins of HTN and chronic kidney disease among children born preterm.^90^ While the ELGAN study sampled only the placental chorion, future cohorts could also consider sampling the maternal decidua in order to compare their epigenetic profiles.

Another limitation is the use of BP measurements from a single study visit, which precludes definitive diagnosis of elevated BP or HTN. Although white coat HTN is associated with increased arterial stiffness and lifetime risk of chronic HTN, reliance on measurements from a single study visit increases the potential for misclassification bias.^91^ Definitive diagnosis of elevated BP or HTN requires consecutive measurements over a longer period of weeks to months.^36^

We were additionally limited by a modest sample size, which limited statistical power to detect associations with the dichotomous outcome of normal versus elevated BP. The smaller sample size also increases the likelihood that the participants differ from the inception cohort, which could introduce bias. The ELGAN cohort, like other extremely preterm birth cohorts, experienced attrition in participants with sociodemographic obstacles to follow-up.^92^ Notably, our sample was demographically similar to the ELGAN participants who completed adolescent follow-up but were not in our sample due to a lack of placental data or no adolescent BP measurement. We also adjusted for sociodemographic confounders, but they are prone to measurement bias and should be tested in other cohorts.

## Conclusion

In the overall sample, we found no association between placental eGA acceleration and adolescent elevated BP. However, among adolescent males born extremely preterm, placental eGA acceleration was associated with higher SBP.

The sex-specific results suggest that placental eGA could provide insights into the developmental origins of disease and might serve as a biomarker to identify subgroups at increased risk for CV and kidney disease.

## Supplementary information

Supplementary information

## Data Availability

The datasets generated during the current study are available from the study investigators on reasonable request and with consideration of participant privacy and ethical restrictions.
