# Supplementary material for: Placental epigenetic age and adolescent blood pressure: the Extremely Low Gestational Age Newborn cohort
Source: Pediatr Res. 2025 May 7;98(5):1780–8. doi: 10.1038/s41390-025-04110-0 (PMC12602336; doi:10.1038/s41390-025-04110-0)
Supplement: Supplementary file 1 — Supplementary information [file 41390_2025_4110_MOESM1_ESM.pdf]

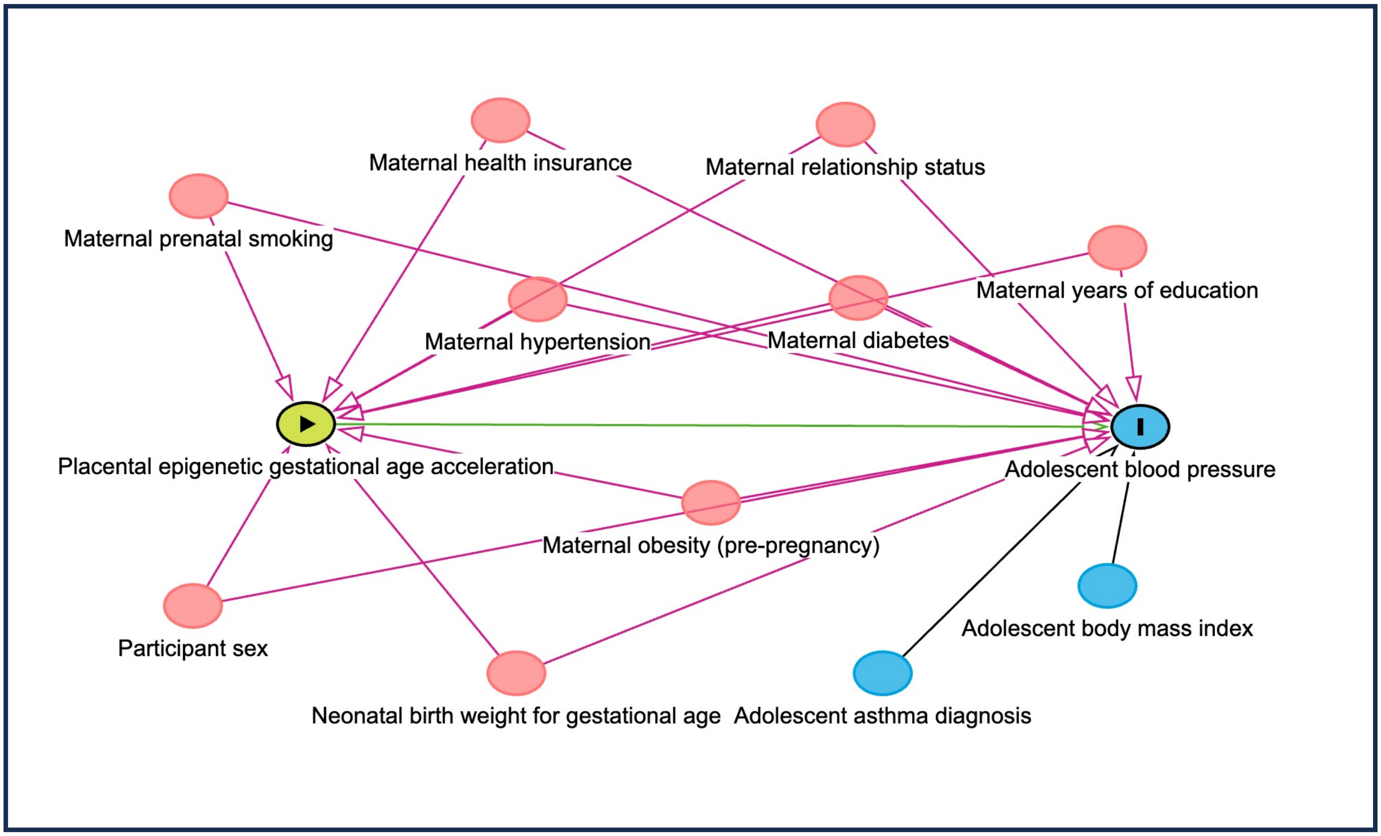

Supplemental Figure S1. Directed acyclic graph. Exposure: placental eGA acceleration at birth; outcome: adolescent SBP. Minimal sufficient adjustment includes maternal factors (prenatal smoking, health insurance, relationship status, hypertension, diabetes, pre-pregnancy obesity, education), participant sex, and neonatal birth weight for gestational age.

Supplemental Table S1. Neonatal, childhood, and maternal characteristics of adolescents 15-18 years old in our sample versus surviving adolescents 15-18 years old in the ELGAN cohort but not in our sample

	In sample (N=193)	Not in sample (N=522)
Maternal characteristics, n (%) or mean (SD)		
Age at delivery (years), mean (SD)	29.5 (7.0)	29.6 (6.6)
BMI (kg/m^2)		
Underweight (<18.5)	13 (6.7%)	41 (7.9%)
Normal (18.5 to <25)	95 (49.2%)	248 (47.5%)
Overweight (25 to <30)	36 (18.7%)	100 (19.2%)
Obese (>=30)	43 (22.3%)	114 (21.8%)
Missing	6 (3.1%)	19 (3.6%)
Education Status		
Less than high school diploma	23 (11.9%)	73 (14.0%)
High school diploma	49 (25.4%)	120 (23.0%)
Some college	44 (22.8%)	117 (22.4%)
Bachelor's degree with or without postgraduate education	74 (38.3%)	212 (40.6%)
Missing	3 (1.6%)	0 (0%)
Public insurance		
Yes	63 (32.6%)	173 (33.1%)
No	130 (67.4%)	349 (66.9%)
Relationship status		
Married or living with partner	157 (81.3%)	427 (81.8%)
Not married or living with partner	36 (18.7%)	95 (18.2%)
Prenatal smoking		
Yes	24 (12.5%)	75 (14.4%)
No	168 (87.0%)	434 (83.1%)
Missing	1 (0.5%)	13 (2.5%)
Diabetes, including gestational, type 1, and type 2		
Yes	16 (8.3%)	34 (6.5%)
No	176 (91.2%)	472 (90.4%)
Missing	1 (0.5%)	16 (3.1%)
Hypertension, including gestational and chronic		
Yes	36 (18.7%)	75 (14.4%)

No	156 (80.8%)	431 (82.6%)
Missing	1 (0.5%)	16 (3.0%)
Participant characteristics, n (%)		
Sex		
Male	100 (51.8%)	261 (50.0%)
Female	93 (48.2%)	261 (50.0%)
Race		
White	125 (64.8%)	341 (65.4%)
Black	52 (26.9%)	116 (22.2%)
Other	16 (8.3%)	55 (10.5%)
Missing	0 (0%)	10 (1.9%)
Adolescent BMI (kg/m^2)		
<=30	154 (79.8%)	396 (75.9%)
>30	25 (12.9%)	51 (9.8%)
Missing	14 (7.3%)	75 (14.3%)
Gestational age (weeks)	26.1 (1.3)	26.1 (1.3)
Birth weight-for-gestational-age z score		
<-2	8 (4.2%)	34 (6.5%)
-2 to <-1	19 (9.8%)	70 (13.4%)
-1 to 1	144 (74.6%)	352 (67.5%)
>1	22 (11.4%)	66 (12.6%)

Supplemental Table S2. Mixed linear regression of placental eGA acceleration on adolescent SBP with sensitivity analysis for cell type proportion*

	B Coefficient	95% CI	P value
Unadjusted model	0.7	-1.2, 2.6	0.47
Adjusted model**	1.1	-0.7, 3.0	0.24
Interaction between eGA acceleration and sex			
Unadjusted model interaction	3.8	0.38, 7.4	0.04
Males	3.2	0.34, 6.1	0.03
Females	-0.6	-2.9, 1.6	0.59
Adjusted model interaction***	4.8	1.4, 8.3	0.01
Males	4.2	1.4, 7.1	0.01
Females	-0.6	-2.8, 1.6	0.61

*all models adjusted for cell type principal components (PCs)

**in addition to adjustment for cell type PCs, also adjusted for SEP, prenatal smoking, maternal BMI>30 kg/m^2, maternal diabetes, maternal hypertension, birth weight for gestational age, and participant sex

***in addition to adjustment for cell type PCs, also adjusted for SEP, prenatal smoking, maternal BMI>30 kg/m^2, maternal diabetes, maternal hypertension, and birth weight for gestational age

Supplemental Table S3. Interaction between sex and placental eGA acceleration on adolescent SBP in adjusted mixed linear regression accounting for adolescent comorbidities

Comorbidity	B Coefficient	95% CI	P value
Adjusted for adolescent BMI>30 kg/m^2*	3.4	0.5, 7.3	0.03
Males	3.2	0.6, 5.9	0.02
Females	-0.6	-2.8, 1.5	0.59
Adjusted for adolescent asthma diagnosis**	4.6	1.1, 8.2	0.01
Males	3.6	0.9, 6.4	0.02
Females	-1.0	-3.3, 1.2	0.40

*adjusted for SEP, prenatal smoking, maternal BMI>30 kg/m^2, maternal diabetes, maternal hypertension, birth weight for gestational age, adolescent BMI >30kg/m^2

**adjusted for SEP, prenatal smoking, maternal BMI>30 kg/m^2, maternal diabetes, maternal hypertension, birth weight for gestational age, adolescent asthma diagnosis
